# Mechanistic, in-silico and *in vitro* studies with nitrofurans reveal potent leishmanicidal activity and inhibition of trypanothione reductase

**DOI:** 10.1016/j.ijpddr.2025.100605

**Published:** 2025-07-31

**Authors:** Julia Andrés-Rodríguez, María-Cristina González-Montero, Nerea García-Fernández, Juan-José Galano-Frutos, Maria-Cristina de Rosa, Patricia Ferreira, María-Yolanda Pérez-Pertejo, Rosa M. Reguera, Rafael Balaña-Fouce, Carlos García-Estrada

**Affiliations:** a Departamento de Ciencias Biomédicas, Facultad de Veterinaria, Universidad de León, Campus de Vegazana s/n, 24007, León, Spain; b Istituto di Scienze e Tecnologie Chimiche “Giulio Natta” (SCITEC) — National Research Council (CNR), Via Largo Francesco Vito 1, 00168, Rome, Italy; c Departamento de Bioquímica y Biología Molecular y Celular — Universidad de Zaragoza, C/ Pedro Cerbuna 12, 50009, Zaragoza, Spain; d Instituto de Biomedicina (IBIOMED), Universidad de León, Campus de Vegazana s/n, 24007, León, Spain

**Keywords:** *Leishmania donovani*, Visceral leishmaniasis, Cyclic nitrofurans, Trypanothione, Trypanothione reductase, Molecular docking

## Abstract

Visceral leishmaniasis caused by *Leishmania infantum* and *Leishmania donovani* is one of the neglected tropical diseases (NTDs) caused by trypanosomatids with treatment options limited to outdated drugs often causing adverse effects and promoting drug resistance. Previous antileishmanial drug discovery campaigns have identified nitroheterocyclic molecules with high efficacy and a high selectivity index. Therefore, we have evaluated on our screening platform of fluorescent *L. donovani* amastigotes, the antileishmanial activity of seven nitrofuran derivatives: furazolidone, nitrofurazone, nitrofurantoin, nifurtimox, 5-nitro-2-furaldehyde diacetate, PYZD-4409 and 5-nitro-2-furonitrile. These compounds showed good efficacy against axenic and intramacrophage amastigotes, most of them showing low cytotoxicity in mammalian cell lines. These nitrofuran derivatives induced reactive oxygen species production in axenic amastigotes and inhibited trypanothione reductase (TryR) either in uncompetitive or competitive manner, thus suggesting that their mechanism of action involves increased oxidative stress caused by an imbalance in redox metabolism. Furazolidone exhibited the most promising antileishmanial profile, and molecular docking analysis revealed consistency with the strongest TryR uncompetitive inhibitory effect, demonstrating its high affinity for an alternative binding site near the substrate (oxidized trypanothione) pocket. Docking results also highlighted PYZD-4409 as the compound with the highest binding affinity, and showed consistency with its competitive inhibition mechanism. Furthermore, similar binding modes identified across *L. donovani* TryR and other homologous proteins suggest the potential broad-spectrum activity of these nitrofuran derivatives, thus underscoring their importance as promising candidates for the development of novel antileishmanial therapies with broad-spectrum applications.

## Graphical abstract

**Figure:**
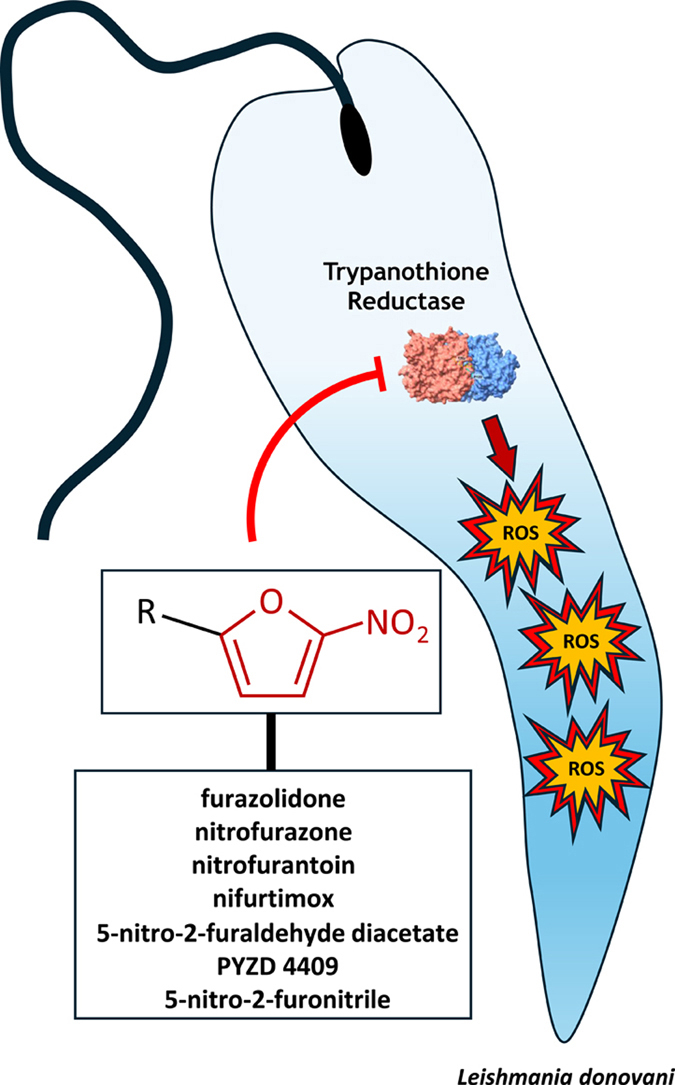

## 1 Introduction

Visceral leishmaniasis (VL) is one of the most prevalent neglected tropical diseases (NTDs) caused by trypanosomatids and is responsible for thousands of deaths annually if left untreated. The etiological agents of VL are *Leishmania donovani* and *Leishmania infantum* in Old World regions, vectored by phlebotomine sandflies (Burza et al., 2018). After various health interventions, VL mortality has been drastically reduced in Asia, but in eastern sub-Saharan Africa it remains a scourge (Wamai et al., 2020). Significant numbers of cases of VL have been reported in central African countries, indicating a possible spread of the disease (Israël et al., 2021; Ngouateu and Dondji, 2022).

When prevention fails and in the absence of a registered human vaccine, VL requires pharmacological treatment, but the current vade mecum is scarce and relies on a handful of drugs, most of them obsolete, with declared toxicity or poor oral bioavailability (van Griensven and Diro, 2019; Reguera et al., 2019; Zhang et al., 2025). Pentavalent antimonials (Sb^V^) have been the drugs of choice since the early decades of the last century, but their massive administration has led to the development of resistance (Croft et al., 2006; Frézard et al., 2009; van Griensven and Diro, 2019), which in the case of India was successfully circumvented with amphotericin B. This polyene macrolide antifungal is highly effective against *Leishmania*, but chemically unstable, thus requiring a cold chain to the point of application and intravenous administration (Hung et al., 1988; Sundar and Chakravarty, 2015; Chakravarty and Sundar, 2019). Miltefosine is a potent antileishmanial drug, but it cannot be administered to pregnant women or women planning to become pregnant due to its teratogenic nature and long half-life (Chakravarty and Sundar, 2019; Roatt et al., 2020; Palić et al., 2022). The other compounds described for clinical use against VL are being replaced due to toxicity, such as pentamidine (van Griensven and Diro, 2019), or must be administered in combination with other drugs to be effective, such as paromomycin (Musa et al., 2012; van Griensven and Diro, 2019).

Due to the undesired side effects of the current antileishmanial drugs and the emergence of resistant pathogens, there is an urgent need for new pharmacological entities for the treatment of VL. Nitroheterocyclic compounds have been repeatedly found in drug discovery campaigns as promising antileishmanial molecules (reviewed by García-Estrada et al., 2023). For example, among the new investigational compounds proposed by Drug for Neglected Diseases Initiative (DNDi) to combat VL, DNDi-0690, a nitroimidazole-based compound, has demonstrated excellent *in vitro* activity against *Leishmania* strains causing both visceral and cutaneous leishmaniasis (Thompson et al., 2017; Van Bocxlaer et al., 2019), and is currently undergoing phase I clinical trials (https://dndi.org/research-development/portfolio/dndi-0690/. Accessed on January 15, 2025). Previous results from our laboratory obtained after the screening of The Anti-Infection Compound Library (reference HY-L002; MedChemExpress) and Prestwick Chemical Library® (Prestwick Chemical Libraries), identified several compounds with a nitroheterocyclic nature with promising results against *Leishmania*. Nifuratel, a compound with a nitrofuran ring in its structure, showed strong antileishmanial efficacy in the order of nM in mouse splenic explant cultures infected with an infrared strain of *L. donovani* and a selectivity index (SI) > 500 against several mammalian cell cultures. *In vivo* results after oral administration showed >70 % reduction of parasite load in spleen and liver and complete remission in a model of cutaneous leishmaniasis caused by *L. major* (Domínguez-Asenjo et al., 2021). Other nitrofuran-derived compounds that have been reported to be effective against *Leishmania* are furazolidone, nitrofurazone and nitrofurantoin derivatives (Neal et al., 1988; Reimão et al., 2010; Zuma et al., 2023).

It is well-established that the mechanism of action of the nitroheterocyclic derivatives lies in their enzymatic activation by nitro reductase (NTR) enzymes present in the parasite, which induce oxidative stress causing parasite death (Hall et al., 2011; Voak et al., 2013; Wyllie et al., 2013, 2016; García-Estrada et al., 2023). In addition, it has been observed for some nitrofuran molecules that they can act as inhibitors of trypanothione reductase (TryR) (García-Estrada et al., 2023; Melcón-Fernández et al., 2023; González-Montero et al., 2024), an enzyme exclusive of trypanosomatids involved in maintaining the sulphur redox balance of the parasite by scavenging oxygen free radicals through the conversion of oxidized trypanothione (T [S]_2_) into its reduced antioxidant form (Dumas et al., 1997; Reguera et al., 2007; Krauth-Siegel and Comini, 2008).

In an attempt to characterize nitro-derived compounds with antileishmanial activity, in the present work we have selected seven compounds containing a nitrofuran group in their structure (furazolidone, nitrofurazone, nitrofurantoin, nifurtimox, 5-nitro-2-furaldehyde diacetate, 1-(3-chloro-4-fluorophenyl)-4-[(5-nitro-2-furanyl)methylene]-3,5-pyrazolidinedione (PYZD-4409) and 5-nitro-2-furonitrile) (Table 1), and tested the *in vitro* leishmanicidal effect on *L. donovani* axenic and intramacrophage amastigotes. The mechanism of action has been determined by measuring the production of reactive oxygen species (ROS) and TryR activity, together with molecular docking studies to evaluate the binding interaction of these nitrofurans with TryR and validate this enzyme as a target for these molecules.

**Table 1:** Overview of the nitrofuran derivatives tested in this work.

Compound Name	PubChem ID	2D Structure
Furazolidone	5323714	
Nitrofurazone	5447130	
Nitrofurantoin	6604200	
Nifurtimox	6842999	
5-Nitro-2-furaldehyde diacetate	7097	
1-(3-chloro-4-fluorophenyl)-4-[(5-nitro-2-furyl)methylene]-3,5-pyrazolidinedione (PYZD-4409)	60111983	
5-Nitro-2-furonitrile	94881	

## 2 Materials and Methods

### 2.1 Chemical reagents and drugs

Seven cyclic nitrofuran compounds (furazolidone, nitrofurazone, nitrofurantoin, nifurtimox, 5-nitro-2-furaldehyde diacetate, 1-(3-chloro-4-fluorophenyl)-4-[(5-nitro-2-furanyl)methylene]-3,5-pyrazolidinedione (PYZD-4409) and 5-nitro-2-furonitrile) (Table 1) were selected to test their activity against *Leishmania* parasites. The compounds were purchased from MedChemExpress (EU Solletuna, Sweden) and dissolved in dimethyl sulfoxide (DMSO) at a stock concentration of 100 mM before use.

### 2.2 Parasites and mammalian cell lines

For efficacy studies, *L. donovani*-iRFP, a genetically modified strain of *L. donovani* LV9 constitutively expressing the gene encoding the infrared fluorescent protein (iRFP) (Melcón-Fernández et al., 2023), was used. This strain was grown as promastigotes in Schneider's insect medium (Sigma-Aldrich, Merck, Darmstadt, Germany) supplemented with 20 % (v/v) foetal bovine serum (FBS) (Gibco, ThermoFisher Scientific, Waltham, MA, USA) and fetal bovine serum cocktail (Gibco, ThermoFisher Scientific, Waltham, MA, USA) and antibiotic cocktail (100 U/mL penicillin and 100 μg/mL streptomycin) (Hyclone, ThermoFisher Scientific, Waltham, MA, USA) at 26 °C until mice were infected (Carballeira et al., 2016). Axenic and intramacrophage amastigotes were isolated from six-to eight-week-old female Balb/c mice (see below) that had been infected intraperitoneally with 1.5 × 10^9^ metacyclic *L. donovani*-iRFP promastigotes. To obtain axenic amastigotes, bone marrow was extracted from the femur and tibia of 8- to 10-week-old infected mice and passed through a 100 μm cell strainer. After centrifugation at 3500 rcf for 10 min at room temperature, cell suspensions were resuspended in medium containing 15 mM KCl; 136 mM KH_2_PO_4_; 10 mM K_2_HPO_4_.3H_2_O; 0.5 mM MgSO_4_.7H_2_O; 24 mM NaHCO_3_; 22 mM glucose; 1 mM glutamine, 1 × RPMI 1640 vitamin mix (Sigma-Aldrich, Merck, Darmstadt, Germany), 10 mM folic acid, 100 mM adenosine, 1 × RPMI amino acid mix (Sigma-Aldrich, Merck, Darmstadt, Germany), 5 mg/mL hemin, 25 mM 2-morpholinoethanesulfonic acid (MES) pH 5.6, supplemented with 10 % FBS (Gibco, ThermoFisher Scientific, Waltham, MA, USA) and antibiotic cocktail, and incubated at 37 °C and 5 % CO_2_. To obtain intramacrophage amastigotes, spleens from infected Balb/c mice were cut into pieces, ground with 2 mg/mL collagenase D (Merck, Darmstadt, Germany) and passed through a 100 μm cell strainer. The splenocyte-enriched cell suspension containing intramacrophage amastigotes was resuspended in RPMI medium (Gibco, Fisher Scientific International Inc., Pittsburgh, PA, USA) supplemented with 20 % FBS, 1 mM sodium pyruvate, 24 mM NaHCO_3_, 2 mM L-glutamine, 1 × RPMI vitamins, 25 mM HEPES pH 7.2 and antibiotic cocktail (Ashok et al., 2016).

For cytotoxicity tests, several mammalian cell lines were used. HepG2 (human hepatocellular carcinoma cell line) and RAW 264.7 (murine macrophage cell line) cells were cultured in Dulbecco's modified Eagle's medium (DMEM) and RPMI, respectively, both supplemented with 10 % FBS and antibiotic cocktail, and incubated at 37 °C and 5 % CO_2_. Primary cell cultures were also used to evaluate cytotoxicity. Mice splenocytes from mouse spleen explants were obtained from healthy, non-infected, mice and were grown as previously described in splenocyte medium (RPMI supplemented with 10 mM HEPES, 1 mM sodium pyruvate, 1xRPMI 1640 vitamin mix, 10 % (v/v) FBS and antibiotic cocktail) (Tejería et al., 2019). Bone marrow-differentiated macrophages (BMDM) were extracted from femur and tibia as indicated elsewhere (Melcón-Fernández et al., 2023) and grown in BMDM medium (RPMI supplemented with 20 % FBS and antibiotic cocktail). BMDM cells were differentiated for one week with 30 % Macrophage and Granulocyte Colony Stimulating Factor (GM-CSF) from L929 cell line.

### 2.3 Experimental animals and ethical statement

Balb/c mice were purchased from Laboratoires Janvier (St Berthevin Cedex, France). The animals were kept under standard housing conditions in the animal house of the University of León with free access to food and water. The animals were handled and humanely slaughtered according to the protocols of the Spanish Law (RD 53/2013) inspired by the European Union Legislation (2010/63/EU) and were approved by the Junta de Castilla y León under the OEBA-ULE-010-2023 authorisation.

### 2.4 Assessment of the *in vitro* and *ex vivo* antileishmanial effect and cytotoxicity

The antileishmanial effect of the nitrofurans selected in this study was tested both *in vitro* on axenic *L. donovani*-iRFP amastigotes and *ex vivo* on intramacrophage amastigotes according to previously published methods (Melcón-Fernández et al., 2023, 2024; Andrés-Rodríguez et al., 2024). Assays were performed in a final volume of 40 μL containing 20,000 axenic amastigotes or a number of *L. donovani*-iRFP-infected murine splenocytes representing 150,000 relative fluorescence units (RFU), in each well of a black 384-well microtiter plate with optical bottom. In addition, 40 μL of one-half serial dilutions of each compound in amastigotes culture medium (for axenic amastigotes) or in supplemented RPMI medium (for intramacrophage amastigotes) were added to each well. Positive (amphotericin B at a final concentration of 18 μM) and negative (0.1 % v/v DMSO) controls were included on each plate. The plates were incubated at 37 °C and 5 % CO_2_ for a maximum of 72 h. Fluorescence emitted by viable *L. donovani*-iRFP was measured on an Odyssey infrared imaging system (Li-Cor, NE, USA).

The *in vitro* cytotoxicity of the selected nitrofurans was evaluated in HepG2, RAW 264.7 cells, splenocytes and BMDM. In each well of a 96-well microtitre plate, 100 μL containing 10,000 HepG2 or RAW 264.7 cells were incubated for 24h at 37 °C and 5 % CO_2_ to allow the cells to settle. For primary cells, 100 μL containing 100,000 BMDM or 1,000,000 splenocytes were added to each well. Subsequently, 100 μM of one-half serial dilutions of each compound diluted in DMEM (for HepG2 cells), RPMI medium (for RAW cells), splenocyte medium (for splenocytes) or BMDM medium supplemented with 10 % GM-CSF (for BMDM), were added to each well. The cytotoxic effect of the compounds was measured after 72 h of incubation at 37 °C and 5 % CO_2_. For this purpose, a 10 % (v/v) of the alamarBlue™ cell viability reagent (Invitrogen, Fisher Scientific International Inc., Pittsburgh, PA, USA) was added, and the reaction was incubated for 4 h at 37 °C and 5 % CO_2_. Fluorescence was measured using a Varioskan Lux spectrophotometer (ThermoFisher Scientific, Waltham, MA, USA). Positive controls consisting of 0.1 % (v/v) H_2_O_2_, and negative controls, consisting of 0.1 % (v/v) DMSO, were also included on each plate.

All experiments were carried out in triplicate and included at least three technical replicates. The percentage of viability for each cell line was plotted against each drug concentration using the nonlinear fit analysis of the Sigma Plot 10.1 statistical package, which provided the EC_50_ (half maximal effective concentration) and CC_50_ (half maximal cytotoxic concentration) values for each compound. The SI was calculated from the CC_50_/EC_50_ ratio.

### 2.5 Ames mutagenicity test

Mutagenic properties of furazolidone and nitrofurazone were tested in TA98 and TA100 *Salmonella typhimurium* strains with and without metabolic activation (S9 mix) using the Ames 384-ISO™ Well Test (EBPI, Burlington, Ontario, Canada), following manufacturers' instructions.

### 2.6 Analysis of ROS production by axenic amastigotes

To assess ROS production in *L. donovani*-iRFP, axenic amastigotes were treated with nitrofurans at the concentration equivalent to their EC_50_ calculated in viability experiments. As positive control of oxidative stress induction, cultures were treated with 0.01 % H_2_O_2_ (v/v), whereas 0.03 % (v/v) DMSO was added as negative control. After an incubation period of 3 h at 37 °C, cells were harvested by centrifugation at 3500 rcf for 10 min, washed in phosphate-buffered saline (PBS), centrifuged again and resuspended in PBS. Cells were labeled with 5 μM 2′,7′-dichlorofluorescein diacetate (DCFH-DA, MedChemExpress) for 30 min at 37 °C. Then, cells were centrifuged at 3500 rcf for 5 min, washed, and resuspended in PBS. The cell suspension was stored at 4 °C until the analysis by flow cytometry, which was performed in a CytoFLEX SRT (Beckman Coulter). The experiment was carried out in triplicate and included three technical replicates.

### 2.7 TryR enzymatic assay

The activity of *L. donovani* TryR was measured in the presence of different concentrations of nitrofurans according to the protocol previously described (Melcón-Fernández et al., 2023). Briefly, 1 × 10^10^ *L. donovani*-iRFP axenic amastigotes were washed twice in PBS and lysed with a solution containing 1 mM EDTA, 40 mM HEPES, 50 mM Tris HCl pH 7.5, 2 % (v/v) Triton X-100 and Pierce™ Protease Inhibitors Mini Tablets (ThermoFisher Scientific, Waltham, MA, USA). Cell extracts were prepared by mechanical disruption of the cells with 0.5 mm diameter glass beads (Merck, Darmstadt, Germany) and the supernatant obtained by centrifugation at 10,000 rcf for 5 min at 4 °C was used to measure TryR activity in 96-well plates. Each well included 2 μL of different concentrations of nitrofurans (from 6.25 μM to 100 μM) diluted in DMSO, 28 μL of TryR assay solution (200 μM NADPH (Alfa Aesar, Fisher Scientific International Inc., Pittsburgh, PA, USA), saturating concentrations of T [S]_2_ (75 μM) (Bachem, Fisher Scientific International Inc., Pittsburgh, PA, USA), 75 μM 5,5′-dithiobis (2-nitrobenzoic acid) (DTNB) (Alfa Aesar, Fisher Scientific International Inc., Pittsburgh, PA, USA) and 50 mM Tris HCl pH 7.5). Cell extracts diluted in Tris HCl pH 7.5 (0.43 μg of total protein in 50 μL) were added to launch the reaction. The negative control consisted of 2.5 % DMSO, whereas the blank reaction included the TryR assay solution without T [S]_2_. As positive control of inhibition activity, 0.1 mM thioridazine (Medchem Express), a well-known inhibitor of TryR (Lo Presti et al., 2015) in DMSO, was used. In addition, assays varying the concentrations of T [S]_2_ (from 25 μM to 150 μM) at different concentrations of nitrofurans (from 6.25 μM to 100 μM) were also performed, and the Lineweaver-Burk double reciprocal plot was used to determine the type of inhibition. To determine steady-state conditions, enzymatic activity was measured at 412 nm (ε = 14,150 M^−1^ cm^−1^) for a period of up to 120 min (with 5 min intervals) at 26 °C in a Varioskan Lux spectrophotometer (ThermoFisher Scientific, Waltham, MA, USA). The experiment was carried out in triplicate and included three technical replicates.

### 2.8 Compounds and proteins 3D structures used in molecular docking

A crystal structure for the putative target of the tested compounds, *L. donovani* TryR (P39050 entry in UniProtKB database), was not available. However, a reliable AlphaFold (AF) model with an average model confidence index (pLDDT) of 97.09 was available and used for docking analysis. Crystal structures of the highly homologous TryR from *L. infantum* (A4HSF7 in UniProtKB), and for other trypanosomatids, such as *Trypanosoma cruzi* (P28593 in UniProtKB) and *Trypanosoma brucei brucei* (Q389T8 in UniProtKB) were also selected for comparison and considered for docking experiments with the nitrofuran derivatives. Sequence identity percentages and backbone root mean square deviation (RMSD) values between these TryRs and the AF model of *L. donovani* TryR are reported in Table 2.

**Table 2:** Comparison between TryR receptors (sequence and structure) modeled by docking.

UniProtKB (source)	Seq. Length (AAs)	Seq. Identity^a^ (%)	Crystal Structure^b^ (PDB ID)	Crystal Resolution (Å)	RMSD vs. *L. donovani* AFmodel^c^ (Å)
P39050 (*L. donovani*)	491	–	not available	–	–
A4HSF7 (*L. infantum*)	491	98.78	4ADW (Baiocco et al., 2013)	3.61	1.37
P28593 (*T. cruzi*)	492	66.67	1BZL (Bond et al., 1999)	2.40	2.19
Q389T8 (*T. brucei brucei*)	492	66.12	2WOW (Patterson et al., 2011)	2.20	2.43

a Sequence identity versus *L. donovani* TryR sequence using ClustalW (v.1.2.4, online) (https://www.ebi.ac.uk/jdispatcher/msa/clustalo. Accessed on 10 October 2024).

b 3D crystal structures were selected primarily for their inclusion of the trypanothione substrate and their good or reasonable resolution.

c Backbone RMSD against the AF model (Jumper et al., 2021) of *L. donovani* TryR (https://alphafold.ebi.ac.uk/entry/P39050. Accessed on 10 October 2024) obtained by the RMSD calculator plugin of the visual molecular dynamics (VMD) software (Humphrey et al., 1996).

The 3D initial structures of the nitrofuran derivatives were downloaded from the PubChem database (Kim et al., 2023), with their respective PubChem IDs and 2D structures given in Table 1. These structures were used without further minimization in the docking runs.

### 2.9 Pocket prediction and cross-docking

Binding pocket predictions were carried out using the PrankWeb online tool (Jendele et al., 2019; Jakubec et al., 2022) before performing docking for the selected TryR structures (Table 2). The PrankWeb server is based on P2Rank, a widely-used machine learning program that leverages evolutionary homology (Krivák and Hoksza, 2018). A series of re-docking tests on crystal structures of ligand-TryR complexes (TryR from *L. infantum* and *T. brucei*) were performed using three docking programs: AutoDock Vina (v.1.2.5) (Trott and Olson, 2010), Ledock (Zhang and Zhao, 2016) and Glide (Schrödinger Release, 2024-1: Glide, Schrödinger, LLC, New York, NY, 2024; Friesner et al., 2004). Results (not shown) revealed that flexible (receptor) docking with AutoDock Vina (v.1.2.5) (Trott and Olson, 2010) most accurately reproduced the crystal ligand-binding interactions (RMSD_lig_ = 0.8 ± 0.3 Å, n = 12) in these two targets, which are highly homologous to *L. donovani* TryR. Therefore, AutoDock Vina (v.1.2.5) was selected to run flexible, local docking on one of the two substrate binding sites for the nitrofurans evaluated in this study. Side-chain rotatable bonds of key residues within the T [S]_2_ binding pocket were allowed to rotate during docking. Flexible docking refers to methods in which the rotatable bonds of both the ligand and the protein receptor are allowed to rotate, enabling exploration of conformational changes during the process. The monomeric structure of *L. donovani* TryR obtained from AF (https://alphafold.ebi.ac.uk/entry/P39050) was used to model the dimeric structure of the holoprotein ―by rotation and superimposition―, using the crystallographic dimeric structure of *L. infantum* TryR as a template. The NADPH cofactor and the T [S]_2_ substrate ― presented in its open form ― were positioned on the dimeric AF-based model to mimic their binding poses as observed in the crystal structure. Three other highly homologous TryRs (PDB entries indicated in Table 2 of the main article) to *L. donovani* TryR were also evaluated as receptor targets in this study, with all structures prepared as detailed below. The crystal structures of *T. brucei* (PDB ID 2WOW) (Patterson et al., 2011) and *T. cruzi* (PDB ID 1BZL) (Bond et al., 1999) exhibit the trypanothione substrate in closed forms (disulfide-bridged), binding differently within the same binding pocket.

**Fig. 1:**
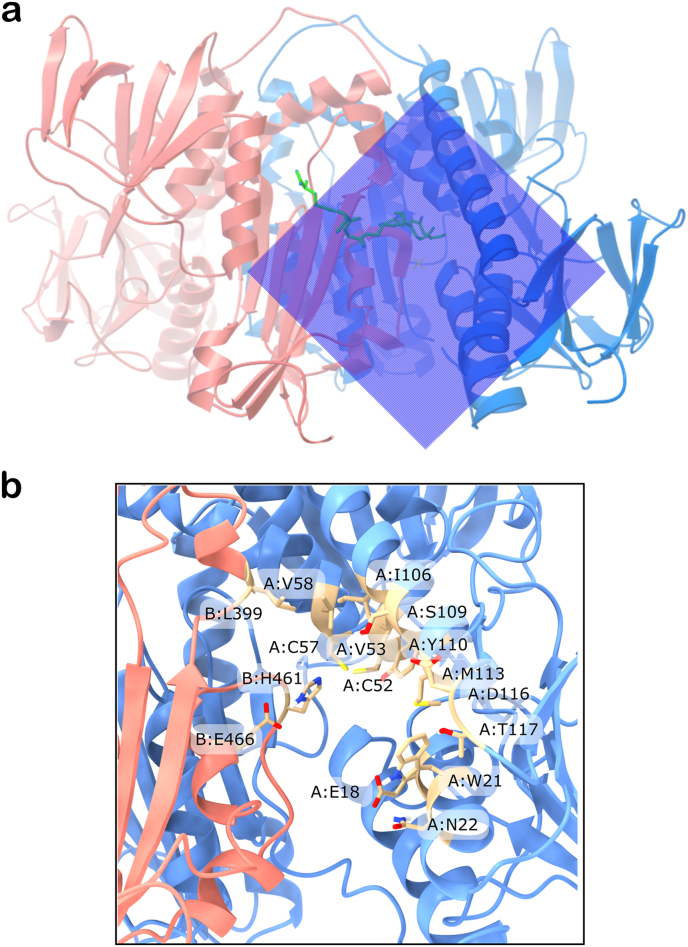
Grid box and flexible residues. a) Cubic grid box of 27,000 Å3 (30 Å × 30 Å × 30 Å) settled for the flexible, local docking, centered on one of the two T [S]_2_ substrate binding pockets of TryR receptors. The AF-based model of *L. donovani* TryR is shown in cartoon representation (chain A in blue and chain B in red salmon), with T [S]_2_ (green stick representation) in its open form enclosed within the grid box. b) Zoomed-in view of the binding site of T [S]_2_ selected for docking runs, showing (in sticks) the residues set as flexible.

The dimeric TryR receptors (from AF or crystal structures) were prepared using ad-hoc Python scripts available within the AutoDockTools suite (ADT) (Morris et al., 2009), a module of the MGLTools suite (The Scripps Research Institute, MGLTools, Version 1.5.7, (2024). https://ccsb.scripps.edu/mgltools/. Accessed on 10 October 2024). Autodock 4 atom types were assigned along with default Gasteiger charges (Gasteiger and Marsili, 1978), and only polar hydrogens were added. A cubic grid box of 27,000 Å3 (30 Å × 30 Å × 30 Å) was centered on the substrate binding site, fully encompassing it (Fig. 1, panel a), while an exhaustiveness parameter of 120 was sufficient for search convergence. The AutoDock Vina scoring function was used to rank the docked poses. The nitrofuran derivatives tested (Table 1) were also prepared through an ad-hoc Python script available within the ADT suite, and their partial charges (not relevant for AutoDock Vina) were anyway retained from the SDF files downloaded from PubChem (Kim et al., 2023). The side-chain rotatable bonds of the following list of residues in the selected TryR receptors (Table 2) were allowed to rotate: chain A: E18, W21, N22, CYS52, V53, C57, V58, I106, S109, Y110, M113, D116 and T117; chain B: L399, H461 and E466 (Fig. 1, panel b). The molecular docking study was conducted under two receptor scenarios, namely: 1) TryR with the FAD cofactor and the coenzyme (NADPH, if present in the original structure) retained in their crystallographic positions, but without T [S]_2_ substrate; 2) TryR with the FAD, NADPH and T [S]_2_ (or FAD and T [S]_2_) retained in their crystallographic positions.

Semi-flexible docking was performed with AutoDock Vina (Trott and Olson, 2010) on the entire dimeric *L. donovani* TryR surface. Semi-flexible docking refers to a method in which the protein receptor is kept rigid while the rotatable bonds of the ligand are allowed to freely rotate. The L. *donovani* TryR and the nitrofuran derivatives were prepared similarly to the procedure described above for flexible local docking, except that flexibility was not allowed for the protein receptor. A cubic grid box of 830,584 Å3 (94 Å × 94 Å × 94 Å) centered on the protein's geometric center (encompassing the entire TryR structure) was settled. The exhaustiveness parameter was increased to 600 to ensure search convergence.

Docking results were visualized and anayzed using both ChimeraX (UCSF ChimeraX, University of California, San Francisco) (Goddard et al., 2018; Meng et al., 2023) and BIOVIA (Dassault Systèmes, Discovery Studio, 2024; San Diego).

## 3 Results

### 3.1 In vitro effect of nitrofurans on L. donovani-iRFP and mammalian cell lines

**Fig. 2:**
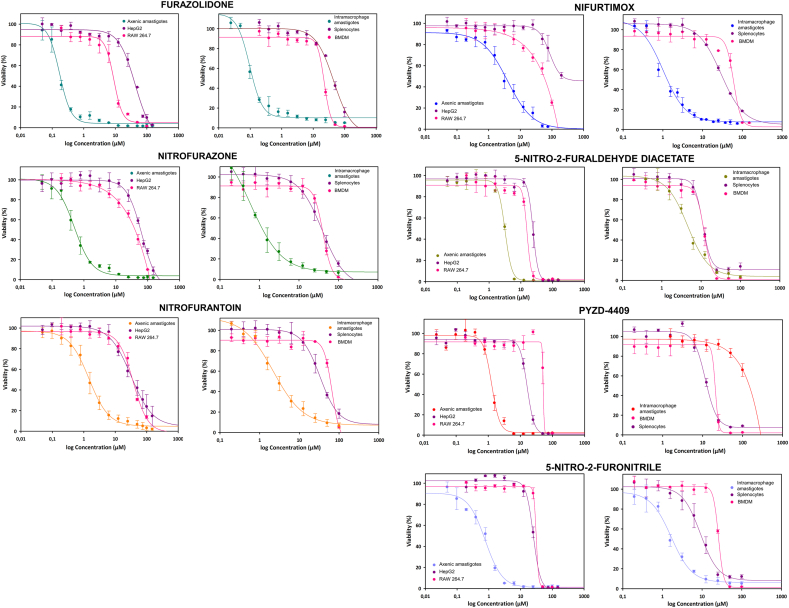
Dose response curves adjusted with the Sigma Plot 10.1 statistical package showing the antileishmanial efficacy and cytotoxicity of furazolidone, nitrofurazone, nitrofurantoin, nifurtimox, 5-nitro-2-furaldehyde diacetate, 1-(3-chloro-4-fluorophenyl)-4-[(5-nitro-2-furanyl)methylene]-3,5-pyrazolidinedione (PYZD-4409) and 5-nitro-2-furonitrile. The left panel of each compound represents the efficacy on L. infantum-iRPF axenic amastigotes and the cytotoxicity on established HepG2 and RAW-264.7 cell lines. Right panels represent the antileishmanial effect on intramacrophage amastigotes and the cytotoxicity assessed on primary macrophages from mouse bone marrow (BMDM) and spleen. The nitrofuran concentrations ranged from 150 μM to 0.049 μM, with exception of nifurtimox (75 μM–0.049 μM) and PYZD-4409 (100 μM–0.024 μM). Dose-response curves were performed plotting the percentage of viability obtained from cells after 72 h of incubation in the presence of the nitrofurans. As negative control 0,1 % (v/v) DMSO was used (100 % viability), whereas 18 μM amphotericin B was used as positive control (0 % viability). Data represent the mean ± standard deviation of three different experiments carried out in triplicate.

In view of our interesting previous results with the nitrofuran nifuratel in *L. infantum* and *L. major* (Domínguez-Asenjo et al., 2021), seven commercial nitrofurans (furazolidone, nitrofurazone, nitrofurantoin, nifurtimox, 5-nitro-2-furaldehyde diacetate, 1-(3-chloro-4-fluorophenyl)-4-[(5-nitro-2-furanyl)methylene]-3,5-pyrazolidinedione (PYZD-4409) and 5-nitro-2-furonitrile) (Table 1), were selected for analysis on our *L. donovani-*iRFP screening platform. Fig. 2 contains the dose/response curves of the seven compounds tested in our study on axenic amastigotes of *L. donovani*-iRFP (left panels) and on mouse splenic explants infected with *L. donovani-*iRFP (right panels). In these experiments, the fluorescence emitted by the *L. donovani*-iRFP parasites measures parasite viability. As negative control, 0.1 % (v/v) DMSO (100 % viability) was used, whereas the positive control consisted of cultures treated with 18 μM amphotericin B, a well-known leishmanicidal agent in clinical use (0 % viability). To evaluate the safety of the tested compounds, we compared the antileishmanial efficacy of the compounds over axenic amastigotes with the cytotoxic effect on established mammalian cell lines; namely HepG2 (human hepatocarcinoma) representing a model to study systemic toxicity, and RAW 264.7 (murine macrophages), the latter representing a model of cells harbouring the parasite (Fig. 2, left panels). Regarding intramacrophage amastigotes, safety was evaluated by comparison of the efficacy of the compounds on the parasites with primary macrophages obtained from both the spleen and bone marrow of uninfected mice (Fig. 2, right panels). Cytotoxicity was determined using the alamarBlue™ cell viability reagent as indicated in the Materials and Methods section. Table 3 summarizes the results obtained with the dose/response curves of the axenic amastigotes of the seven nitrofurans and their corresponding cytotoxicity in HepG2 and RAW 264.7 cell cultures using the SigmaPlot 11.0 software package, which provides the EC_50_ and CC_50_ values, respectively.

**Table 3:** Results of EC_50_, CC_50_ and SI for the nitrofuran derivatives tested in *L. donovani-*iRFP axenic amastigotes, HepG2 and RAW 264.7 cells.

Tested Compound	*L. donovani*-iRFP axenic amastigotes	HepG2 cells	RAW 264.7 cells	SI_1_	SI_2_
	EC_50_ (μM)	CC_50_ (μM)	CC_50_ (μM)		
Furazolidone^a,b^	0.17 ± 0.01	42.46 ± 6.81	21.58 ± 0.69	249.76	126.94
Nitrofurazone^a,b^	0.49 ± 0.03	65.50 ± 9.67	39.61 ± 7.91	133.67	80.84
Nitrofurantoin^a^	1.46 ± 0.08	32.05 ± 4.58	37.20 ± 7.05	21.95	25.48
Nifurtimox^a^	3.96 ± 0.37	84.92 ± 12.38	50.53 ± 2.25	21.44	12.76
5-nitro-2-furaldehide diacetate	3.48 ± 0.09	24.08 ± 0.59	15.62 ± 1.27	6.92	4.49
PYZD-4409^a^	1.27 ± 0.04	16.27 ± 0.56	51.73 ± 8.03	12.81	40.73
5-nitro-2-furonitrile^a^	0.70 ± 0.05	21.69 ± 0.39	31.21 ± 5.04	30.99	44.59

**Note:** SI_1_ calculated between axenic amastigotes and HepG2 cells. SI_2_ calculated between axenic amastigotes and RAW 264.7 cells. A high SI (>1) indicates that the compound is more selective for the desired effect than for general cytotoxicity. ^a^Hit (EC_50_ ≤ 10 μM and SI ≥ 10) and ^b^Lead (EC_50_ ≤ 1 μM and SI ≥ 50) compounds according to criteria proposed for visceral leishmaniasis (Don and Ioset, 2014; Katsuno et al., 2015).

All seven compounds studied had a very high efficacy, with EC_50_ values ranging from 3.96 μM (nifurtimox) to 0.17 μM (furazolidone). These data, together with low-moderate cytotoxicity in both cell lines, yielded SI values > 1 for all compounds, with furazolidone (SI = 249.8 in HepG2 cells) and nitrofurazone (SI = 133.7 in HepG2 cells) standing out from the rest of compounds.

Results from the dose/response curves of the seven nitrofurans with the intramacrophage amastigotes and cytotoxicity in primary cultures of splenocytes and BMDM is shown in Table 4.

**Table 4:** Results of EC_50_, CC_50_ and SI for the nitrofuran derivatives tested in *L. donovani-*iRFP intramacrophage amastigotes, splenocytes and BMDM.

Tested Compound	*L. donovani-* iRFP intramacrophage amastigotes	Splenocytes	BMDM	SI_1_	SI_2_
	EC_50_ (μM)	CC_50_ (μM)	CC_50_ (μM)		
Furazolidone^a,b^	0.09 ± 0.004	44.76 ± 3.93	22.39 ± 1.01	497.33	248.78
Nitrofurazone^a,b^	0.49 ± 0.11	38.92 ± 6.32	40.55 ± 1.97	79.43	82.76
Nitrofurantoin^a^	2.10 ± 0.26	33.19 ± 3.50	56.31 ± 9.06	15.80	26.81
Nifurtimox^a^	1.00 ± 0.07	37.80 ± 6.12	60.22 ± 4.79	37.80	60.22
5-nitro-2-furaldehide diacetate	4.60 ± 0.26	11.02 ± 0.28	11.92 ± 0.46	2.40	2.59
PYZD-4409	>100	11.73 ± 0.50	20.77 ± 6.40	0.12	0.21
5-nitro-2-furonitrile	1.45 ± 0.11	8.49 ± 0.52	25.82 ± 0.60	5.86	17.81

**Note:** SI_1_ calculated between intramacrophage amastigotes and splenocytes. SI_2_ calculated between intramacrophagic amastigotes and BMDM. A high SI (>1) indicates that the compound is more selective for the desired effect than for general cytotoxicity. ^a^Hit (EC_50_ ≤ 10 μM and SI ≥ 10) and ^b^Lead (EC_50_ ≤ 1 μM and SI ≥ 50) compounds according to criteria proposed for visceral leishmaniasis (Don and Ioset, 2014; Katsuno et al., 2015).

The EC_50_ values after the non-linear fit of the dose/response results provided by SigmaPlot 11.0 showed that, with exception of PYZD-4409, which unexpectedly showed an EC_50_ > 100 μM and unacceptable SI < 1, the other six nitrofurans retained high antileishmanial efficacy to kill the intracellular form of *Leishmania* within the low micromolar and nanomolar range (from 4.60 μM in the case of 5-nitro-2-furaldehide diacetate, to 0.09 μM for furazolidone). In addition, moderate toxicity was found across the primary macrophage cultures, thus leading to the SI value of 497.3 for furazolidone using splenic macrophages and 248.8 using BMDM. According to the criteria proposed to identify hits and leads for visceral leishmaniasis (Don and Ioset, 2014; Katsuno et al., 2015), furazolidone and nitrofurazone qualify as lead candidates. Consistency shown across both forms of amastigotes, axenic and intramacrophagic, suggests a promising avenue for the development of potential antileishmanial therapies.

### 3.2 Mutagenic properties of furazolidone and nitrofurazone

Due to the therapeutic potential of furazolidone and nitrofurazone observed in the *in vitro* experiments reported above, further safety assays were conducted. Mutagenicity of these two compounds was tested using a 384-well microplate Ames test (see Materials and Methods) with TA98 (detection of frameshift mutations) and TA100 (detection of base-pair mutations) *S*. *typhimurium* strains with and without metabolic activation. Considering the EC_50_, five concentrations were tested for furazolidone (from 0.025 μM to 10 μM) and nitrofurazone (from 0.125 μM to 50 μM). Results (Table 5) indicate that furazolidone behaves as a mutagen from 0.025 μM to 0.1 μM, producing cytotoxic effects at higher concentrations. Interestingly, metabolic activation prevents mutagenicity of this compound. On the other hand, nitrofurazone only provided mutagenic effects in the TA100 strain with and without metabolic activation at concentrations ranging from 0.25 μM (or 0.5 μM in the presence of S9 mix) to 5 μM, showing cytotoxic effects at 50 μM. These results indicate a different mechanism of mutagenesis for furazolidone and nitrofurazone (see Discussion).

**Table 5:** Mutagenic activity of furazolidone and nitrofurazone in *S. typhimurium* TA98 and TA100 in the presence and absence of metabolic activation (S9 mix).

Compound	Concentration (μM)	Mutagenic effect^a^			
		TA98	TA98-S9	TA100	TA100-S9
Furazolidone	0.025	+ (0.01)	–	+ (0.001)	–
	0.05	+ (0.05)	–	+ (0.001)	–
	0.1	+ (0.05)	–	+ (0.05)	–
	1.00	-b	–	-b	–
	10.0	-b	–	-b	–
Nitrofurazone	0.125	–	–	–	–
	0.25	–	–	+ (0.01)	–
	0.5	–	–	+ (0.001)	+ (0.05)
	5.0	–	–	+ (0.05)	+ (0.001)
	50.0	–	–	-b	-b

a Positive (+) and negative (−) mutagenic effect and significance level (0.05, 0.01 and 0.001) according to the manufacturers' instructions provided in the Ames 384-ISO™ Well Test kit (EBPI, Burlington, Ontario, Canada).

b Cytotoxic effect.

### 3.3 Nitrofurans induce ROS production in L. donovani-iRFP

**Fig. 3:**
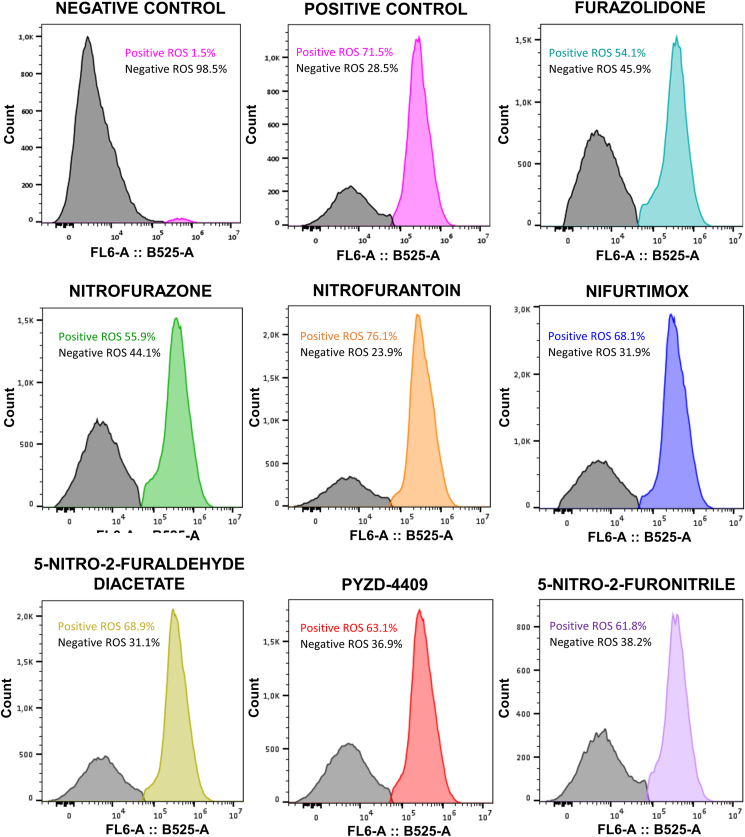
Representative flow cytometry graphs showing ROS production by the axenic amastigote cultures of *L. donovani*-iRFP labeled with DCFH-DA after the addition of 0.01 % (v/v) H_2_O_2_ (positive control), 0.03 % (v/v) DMSO (negative control), 0.17 μM furazolidone, 0.49 μM nitrofurazone, 1.46 μM nitrofurantoin, 3.96 μM nifurtimox, 3.48 μM 5-nitro-2-furaldehyde diacetate, 1.27 μM 1-(3-chloro-4-fluorophenyl)-4-[(5-nitro-2-furanyl)methylene]-3,5-pyrazolidinedione (PYZD-4409) and 0.70 μM 5-nitro-2-furonitrile. Histograms represent the distribution of fluorescence intensity for FL6-A: B525-A. Results show the mean values of three independent experiments with three technical replicates each.

In order to characterize the mechanism of action of the seven nitrofurans under study, the ability of these molecules to generate ROS in *L. infantum*-iRFP axenic amastigotes was tested as indicated in Materials and Methods section. Furazolidone, nitrofurazone, nitrofurantoin, nifurtimox, 5-nitro-2-furaldehyde diacetate, PYZD-4409, and 5-nitro-2-furonitrile were added to cultures of axenic amastigotes at the EC_50_ calculated in previous experiments (Table 3) and incubated for 3 h. Positive (0.01 % v/v H_2_O_2_) and negative (0.03 % v/v DMSO) controls were included, and cells were labeled with DCFH-DA for flow cytometry analysis of ROS. Graphs (Fig. 3) depict distinct peaks corresponding to the stressed and unstressed populations, and clearly indicate that all nitrofurans generated ROS in *L. donovani*-iRFP amastigotes. Nitrofurantoin provided the highest percentage of the stressed population (76.1 ± 11.6 %), whereas furazolidone gave rise to the lowest percentage of the stressed population (54.1 ± 5.8 %).

### 3.4 Nitrofurans behave as TryR inhibitors

**Fig. 4:**
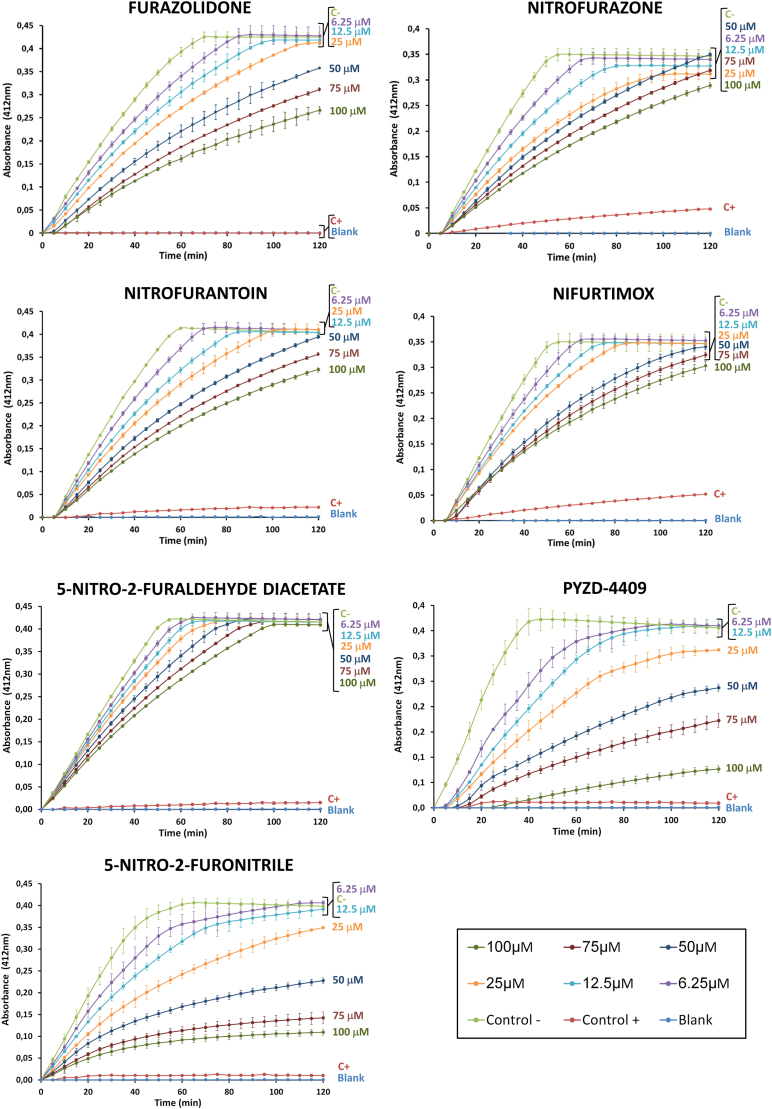
Time-dependent *in vitro* inhibition of *L. donovani* TryR by different concentrations (6.25 μM–100 μM) of furazolidone, nitrofurazone, nitrofurantoin, nifurtimox, 5-nitro-2-furaldehyde diacetate, PYZD-4409, and 5-nitro-2-furonitrile using 0.075 mM T [S]_2_ and 0.20 mM NADPH. Thioridazine (0.1 mM) was used as a positive control (C+), whereas 2.5 % (v/v) DMSO was used as negative control (C-), the latter showing a specific enzymatic activity of 1.83 × 10^−2^ μmol/mg · min. (ΔA/t = ɛ · d · c; ɛ = 14.150 M^−1^ cm^−1^; d = 0.34 cm). Results show the mean values ± SD of three independent experiments with three technical replicates each.

To determine whether the increase in ROS induced by nitrofurans tested above was a consequence of the inhibition of TryR, *L. donovani*-iRFP protein extracts were incubated in the presence of different concentrations (6.25–100 μM) of furazolidone, nitrofurazone, nitrofurantoin, nifurtimox, 5-nitro-2-furaldehyde diacetate, PYZD-4409 and 5-nitro-2-furonitrile, and in *vitro* TryR activity was measured as indicated in the Materials and Methods section. At saturating concentrations of T [S]_2_ (0.075 mM) and NADPH (0.20 mM) the seven nitrofurans were able to reduce TryR activity, PYZD-4409 and 5-nitro-2-furonitrile being the most potent inhibitors (Fig. 4).

**Fig. 5:**
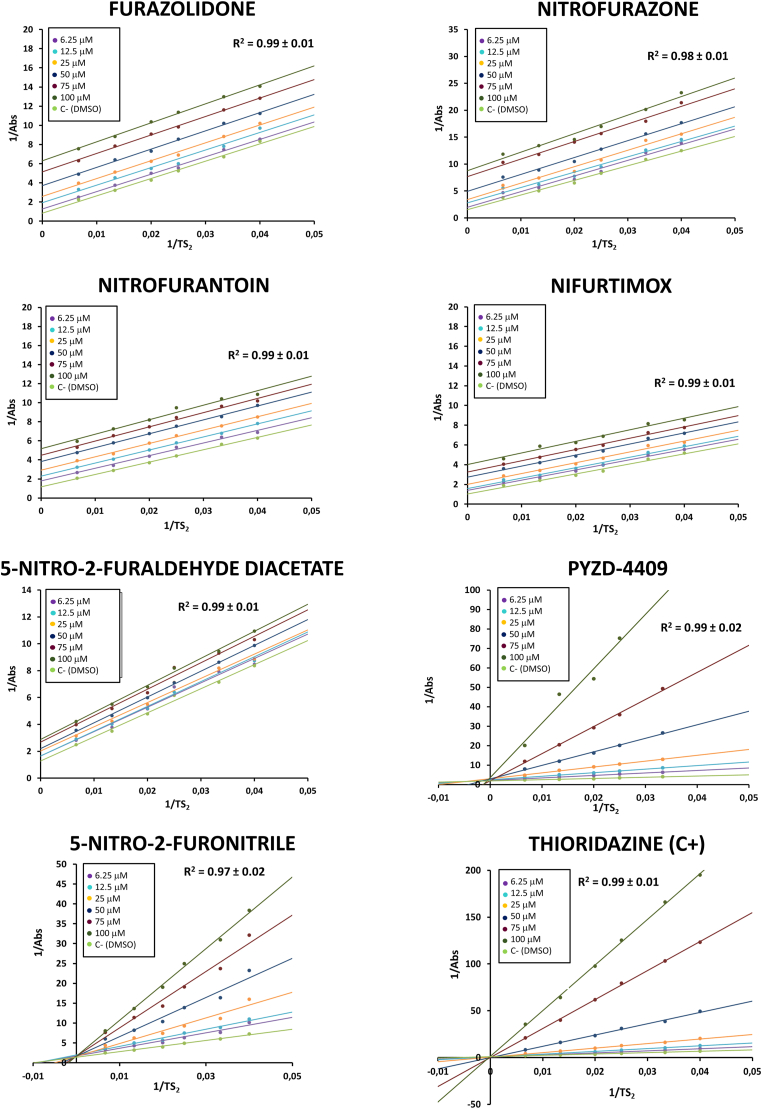
Lineweaver-Burk plots of the inhibition of furazolidone, nitrofurazone, nitrofurantoin, nifurtimox, 5-nitro-2-furaldehide diacetate, PYZD-4409, 5-nitro-2-furonitrilo and thioridazine (positive control) on *L. donovani* TryR. Double reciprocal plots were generated by varying the concentrations of T [S]_2_ (from 25 μM to 150 μM) at different concentrations of each nitrofuran or thioridazine (from 6.25 μM to 100 μM) at a fixed concentration of NADPH (0.20 mM). DMSO (2.5 % v/v) was used as negative control. Results show the mean values of three independent experiments performed in triplicate.

The type of enzyme inhibition and the *K*_i_ value of the seven nitrofurans used in the study was assessed varying the concentrations of T [S]_2_ (from 25 μM to 150 μM) at different concentrations of nitrofurans (from 6.25 μM to 100 μM). The Lineweaver-Burk double reciprocal plot (Fig. 5) showed an uncompetitive inhibition pattern for furazolidone, nitrofurazone, nitrofurantoin, nifurtimox and 5-nitro-2-furaldehide diacetate, whereas PYZD-4409 and 5-nitro-2-furonitrile inhibited *L. donovani* TryR in a competitive manner. The assay was validated with thioridazine, a well-known potent inhibitor of TryR (Lo Presti et al., 2015), which behaved as competitive inhibitor of TryR (Fig. 5).

Table 6 summarizes the inhibitory effect of the seven nitrofurans under study on TryR, their corresponding inhibition mechanism, and the *K*_i_ values. PYZD-4409 was the most potent competitive TryR inhibitor with a *K*_i_ value in the low micromolar range (like thioridazine), thus suggesting a higher affinity for TryR than the other nitrofurans. Furazolidone also showed low *K*_i_ values, although it followed an uncompetitive mode of inhibition.

**Table 6:** Type of inhibition of nitrofurans on TryR and their kinetic parameters. The equation used to calculate *K*_i_ is indicated in the table.

Compound	*K*_i_ (μM)	${K_{m}}_{ap}$	${V_{\max}}_{ap}$	Type of inhibition	Equation
Furazolidone	13.71 ± 2.76	↓	↓	Uncompetitive	KMap=KM1+[I]ki
Nitrofurazone	25.81 ± 4.83	↓	↓	Uncompetitive	
Nitrofurantoin	31.25 ± 4.94	↓	↓	Uncompetitive	
Nifurtimox	33.52 ± 6.93	↓	↓	Uncompetitive	
5-nitro-2-furaldehide diacetate	77.19 ± 21.90	↓	↓	Uncompetitive	
PYZD-4409	7.63 ± 2.56	↑	=	Competitive	KMap=KM×(1+[I]ki)
5-nitro-2-furonitrile	24.78 ± 1.43	↑	=	Competitive	
Thioridazine (C+)	5.79 ± 0.82	↑	=	Competitive	

**Note:** (↑) *K*_M__ap_ > *K*_M_ or *V*_max__ap_ > *V*_max_; (↓) *K*_M ap_ < *K*_M_ or *V*_max ap_ < *V*_max_; (=) *K*_M ap_ = *K*_M_ or V_max ap_ = V_max_.

### 3.5 Predicted binding modes of tested nitrofurans on TryR structures

**Fig. 6:**
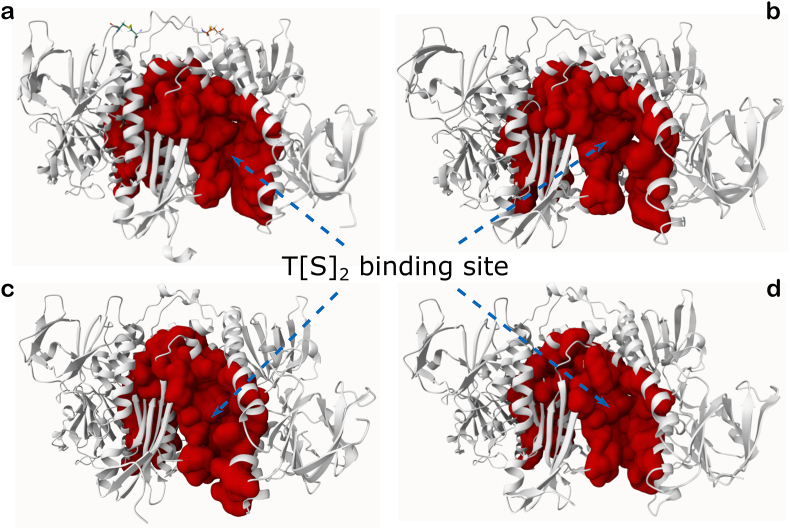
Top-ranked predicted binding pocket by PrankWeb server. Predictions made for: a) the AF-based model of *L. donovani* TryR; b) the crystal structure (PDB ID 4ADW) of *L. infantum*; c) the crystal structure (PDB ID 2WOW) of *T. brucei*; d) the crystal structure (PDB ID 1BZL) of *T. cruzi*. The pockets, represented as red surface areas, encompass both T [S]_2_ binding sites and the internal cavity that connect them. In these representations, only one of the T [S]_2_ binding sites is visible (the other is positioned on the opposite side, facing away), and it is highlighted by blue arrows.

**Fig. 7:**
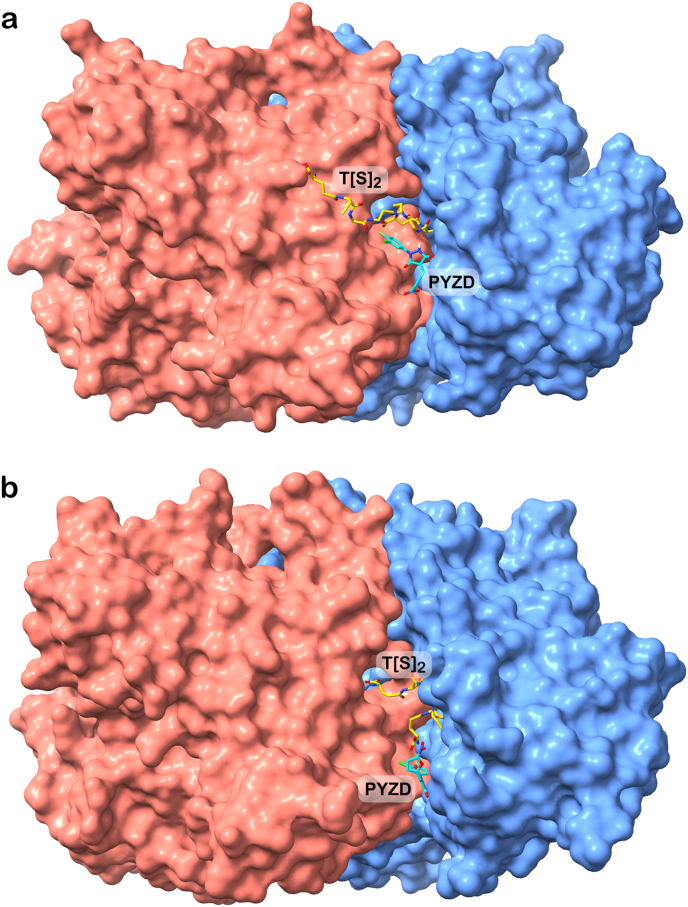
Lowest-energy binding poses as predicted by AutoDock Vina of PYZD-4409 under receptor scenario 2 (with T [S]_2_ in the substrate binding site). a) Best docked pose of PYZD-4409 on the dimeric *L. donovani* TryR, where T [S]_2_ adopts an open conformation (adopted from the *L. infantum* TryR structure in PDB ID 4ADW). b) Best docked pose of PYZD-4409 on the dimeric *T. cruzi* TryR, where T [S]_2_ adopts a closed (disulfide bridged) conformation (PDB ID 1BZL). In both structures, chain A is represented as a blue surface, and chain B as a red surface. Compound PYZD-4409 and T [S]_2_ substrate are shown in stick representation with atoms colored as follows: nitrogen in blue, oxygen in red, fluorine in green, chlorine in pale green, and carbon in yellow (T [S]_2_) or cyan (PYZD-4409).

The cross-docking experiments presented in this work were focused on only one of the two T [S]_2_ substrate binding sites of TryR. This choice was based on the following considerations: 1) several nitrofuran compounds evaluated exhibited competitive inhibition of *L. donovani* TryR in assays with varying T [S]_2_ concentrations, indicating that these compounds may interact with one or both of the substrate binding sites; 2) the PrankWeb server consistently identified ―across all evluated homologous TryR targets ― a prominent superpocket as the top-ranked result encompassing both T [S]_2_ binding sites and an internal cavity connecting them (Fig. 6, Fig. 7). TryR receptors are dimeric structures with global cyclic-C2 symmetry, implying that the two T [S]_2_ binding sites are structurally equivalent.

**Fig. 8:**
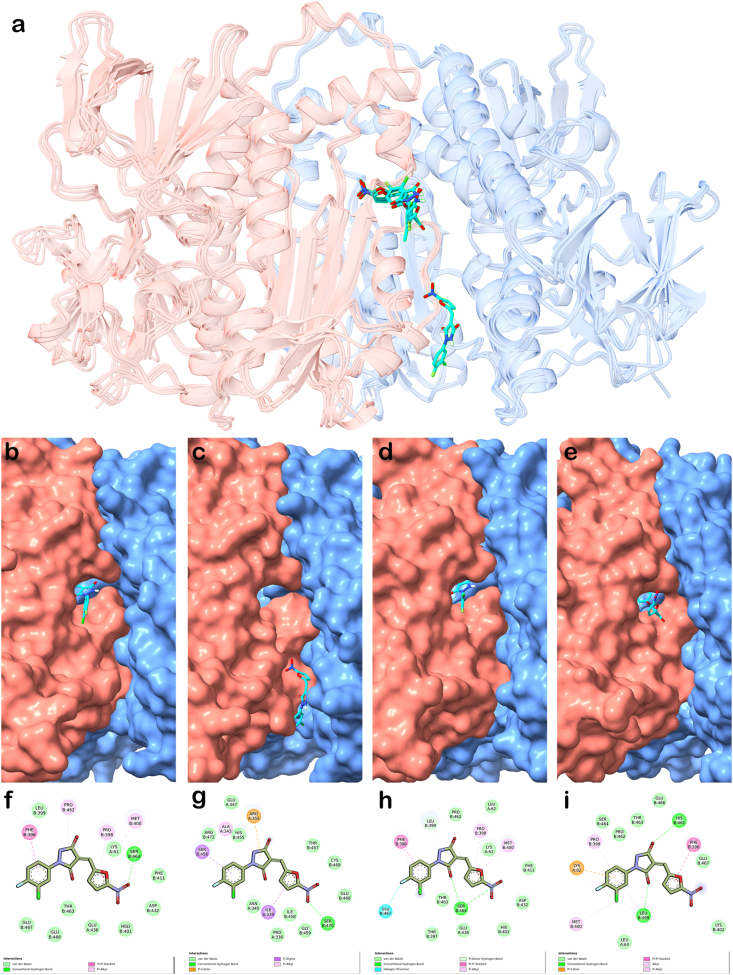
AutoDock Vina-predicted binding poses of PYZD-4409 on TryR and interaction maps for docking scenario 1 as generated by Discovery Studio, version 2024.3 (BIOVIA). a) Overlapped poses (in stick representation, with carbon atoms colored in cyan, nitrogens in blue, oxygens in red, fluorine in green and chlorine in pale green) on the four homologous TryR targets used in this study. b-e) Individual lowest-energy binding modes of PYZD-4409 (in stick representation) on TryR (in surface representation) from *L. donovani*, *L. infantum*, *T. brucei* and *T. cruzi*, respectively. Panels f–i) display the interaction maps corresponding to the binding modes shown in panels b–e.

Under receptor scenario 1 - T [S]_2_ removed from TryR (see Materials and Methods) - docking results showed that compound PYZD-4409 clearly exhibited the top poses with the lowest estimated binding energy (highest affinity) among all the nitrofuran derivatives across the four TryRs evaluated (estimated binding energies ranging from −7.4 to −8.5 kcal/mol, Table 7), with a difference exceeding 1.0 kcal/mol in absolute value compared to the others. The binding poses and interaction maps highlighting key amino acids involved, are shown in Fig. 8a. The lowest-energy binding modes for this compound were observed – among the four receptors – for *L. donovani* (−8.5 kcal/mol) and *T.brucei* (−8.1 kcal/mol) TryRs (Table 7). In both cases, PYZD-4409 binds similarly at the entrance of the cavity connecting the two substrate binding pockets (Fig. 8, panels b and d), positioning its halogen atoms toward the T [S]_2_ binding pocket. Key interacting residues identified in *L. donovani* included F396, P398, M400, P462, S464 and E467 of chain B (Fig. 8, panels f and h). Similarly, for *T. cruzi* TryR, the lowest-energy pose (−7.4 kcal/mol) was also located at the above mentioned cavity entrance, but adopting a distinct compound orientation (Fig. 8, panel e). Key interactions were identified with L62 of chain A, and F396, P398, L399, M400 and H461 of chain B (Fig. 8, panel i). In contrast, steric hindrances at the cavity entrance of *L. infantum* TryR, prevented PYZD-4409 from binding at the same site as in the other TryRs. Instead, the compound adopted an alternative binding position that might block T [S]_2_ access to the active site (Fig. 8, panels c and g). Moreover, docking results on 5-nitro-2-furonitrile, the other competitive inhibitor, revealed a preferential binding position similar to that exhibited by PYZD-4409 on *L. infantum* TryR (Fig. 8, panel c), with slightly different orientations across the four TyrRs (data not shown). However, the associated binding energies were less favourable (Table 7), and this difference – compared to PYZD-4409 – correlates with its higher *K*_i_, reflecting approximately three-fold lower affinity.

**Table 7:** Summary of AutoDock Vina results for nitrofuran derivatives docking (local and flexible) to TryR. Predicted binding energies are reported in kcal/mol.

TryR source	T [S]_2_^a^	Uncompetitive Inhibition^b^					Competitive Inhibition^b^	
		Furazolidone	Nitrofurazone	Nitrofurantoin	Nifurtimox	5-nitro-2-furaldehide diacetate	PYZD-4409	5-nitro-2-furonitrile
*L. donovani*	–	‒6.63	‒6.84	‒6.83	‒6.35	‒6.28	‒8.52	‒5.15
	+	‒6.64	‒6.91	‒6.84	‒6.57	‒6.25	‒8.03	‒5.28
*L. infantum*	–	‒5.57	‒5.88	‒6.26	‒6.24	‒5.24	‒7.48	‒4.46
	+	‒5.58	‒5.82	‒6.26	‒6.26	‒5.30	‒7.60	‒4.54
*T. brucei*	–	‒5.89	‒5.72	‒6.17	‒6.37	‒5.56	‒8.08	‒4.55
	+	‒6.03	‒6.28	‒6.51	‒6.41	‒5.77	‒8.64	‒4.94
*T. cruzi*	–	‒5.73	‒5.63	‒6.23	‒5.92	‒5.23	‒7.35	‒4.84
	+	‒5.31	‒5.21	‒5.81	‒5.45	‒4.93	‒7.15	‒4.58

a Docking scenarios according to the presence (+) or absence (−) of T [S]_2_ bound to TryR receptors.

b Type of enzymatic inhibition observed (see Subsection 3.3) for the nitrofuran derivatives tested.

Docking performed under receptor scenario 2 – with T [S]_2_ retained in TryR structure – revealed that, for *L. donovani* and *T. cruzi* TryRs, the presence of T [S]_2_ prevents PYZD-4409 from binding at the cavity entrance, as observed under docking scenario 1. The estimated binding energies of the new poses (Fig. 7, panels a and b, respectively) were less favourable, indicating lower affinity, compared to those predicted in the absence of T [S]_2_ (Table 7).

**Fig. 9:**
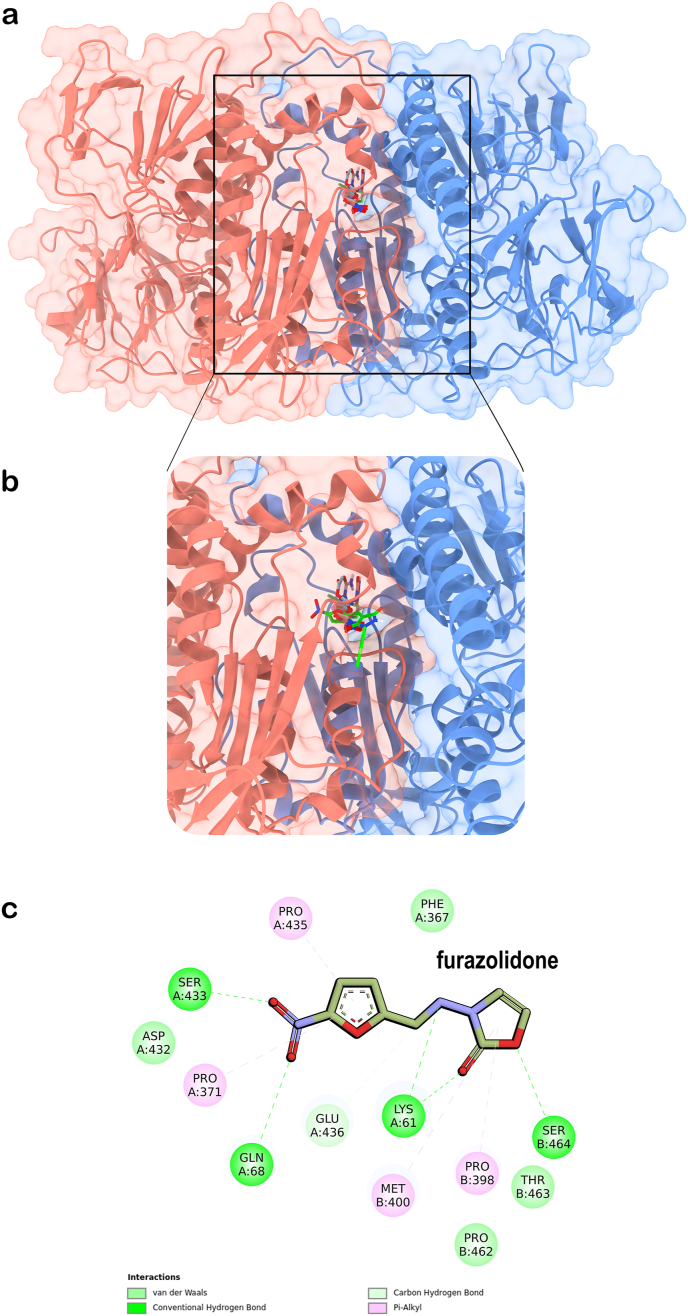
Overlapped lowest-energy poses of furazolidone, nitrofurazone, nitrofurantoin, nifurtimox and 5-nitro-2-furaldehyde diacetate on the dimeric *L. donovani* TryR, and interaction map for furazolidone. a) Results from rigid docking performed on the entire dimeric protein surface, under receptor scenario 1 (with T [S]_2_ removed from TryR structure, see Materials and Methods). The binding energies calculated by AutoDock Vina range from −6.4 to −7.5 kcal/mol. TryR chain A is represented as blue ribbons with a semi-transparent surface, and chain B as red ribbons and a semi-transparent surface. The compounds bound within the internal cavity connecting the two T [S]_2_ substrate binding sites are shown in stick representation, with atoms colored as follows: nitrogen in blue, oxygen in red, and carbon in distinct colors. b) A zoomed-in view of the compounds' binding, including PYZD-4409 (also in stick representation, with carbon atoms in green) for comparison purposes, as bound to *L. donovani* TryR (at the entrance of the mentioned cavity) under flexible docking and receptor scenario 1 (see Fig. 8). c) Interaction map as generated by Discovery Studio, version 2024.3 (BIOVIA), illustrating the binding mode of furazolidone within the mentioned protein internal cavity. The legend below the map specifies the colors used to represent the main identified interactions with protein residues.

The crystal binding modes of the two TryR forms – the open form, observed in *L. donovani* and modeled using the *L. infatum* TryR crystal structure (Fig. 7, panel a), and the closed form, present in the *T. cruzi* TryR crystal structure (Fig. 7, panel b) – either blocked or partially occupied the compound's preferred binding site identified in the absence of T [S]_2_ (Fig. 8, panel b).Semi-flexible docking (see Materials and Methods section) was conducted for furazolidone, nitrofurazone, nitrofurantoin, nifurtimox and 5-nitro-2-furaldehyde diacetate on the entire surface of the AF-based model of *L. donovani* TryR. The best (lowest-energy) poses obtained differ from those identified through flexible docking localized specifically within the T [S]_2_ binding pocket (receptor binding scenario 1: without T [S]_2_). The compound poses in semi-flexible docking are located deeper within the internal cavity connecting the two T [S]_2_ substrate binding sites (Fig. 9), contrasting with their positions – along with that of PYZD-4409 (Fig. 9, panel b) – near the cavity entrance, as observed in flexible local docking. The estimated binding energies of the best poses obtained through semi-flexible docking ranged from −6.4 kcal/mol (5-nitro-2-furaldehyde diacetate) to −7.5 kcal/mol (furazolidone; Fig. 9 panel c), indicating slightly higher affinities compared to those observed under flexible local docking for *L. donovani* TryR (see Table 7).

## 4 Discussion

Faced with the need to find new antileishmanial compounds, nitroheterocyclic molecules have emerged as promising molecules against trypanosomatids-borne diseases (García-Estrada et al., 2023). In search for new drugs for the treatment of VL, in this work we have tested the efficacy and cytotoxicity of seven nitrofurans, a class of nitroheterocyclic synthetic molecules introduced in the mid-20th century as potential allies in the battle against antibiotic-resistant bacteria (Le and Rakonjac, 2021). These Schiff base derivatives of 5-nitrofuraldehyde exhibit broad-spectrum redox-active properties, and for those including a hydrazone moiety, the latter contributes to the overall chemical stability of the nitrofuran ring due to its zwitterionic properties and is also responsible for their antipathogenic activity (Spain, 1995; Trukhacheva et al., 2005; Zuma et al., 2019). As shown in Fig. 2 and Table 3, Table 4, furazolidone, nitrofurazone, nitrofurantoin, nifurtimox, 5-nitro-2-furaldehyde diacetate, PYZD-4409 and 5-nitro-2-furonitrile, showed good efficacy against axenic and intramacrophage *L. donovani-*iRFP amastigotes (only PYZD-4409 provided low efficacy in intramacrophage amastigotes) and exhibited low cytotoxicity in mammalian cell lines. The discrepancy between the potency of PYZD-4409 against axenic or intracellular amastigotes may be attributed to an insufficient uptake of this molecule by the host macrophage, thereby preventing the drug-target interaction at therapeutic concentrations within the mammalian cell. A similar phenomenon has been observed with fexinidazole, another nitroheterocyclic compound of the nitroimidazole family (Andrés-Rodríguez et al., 2024).

Although furazolidone does not exhibit the most favourable toxicity profile toward mammalian cells among the nitrofuran derivatives evaluated (Table 3, Table 4), its markedly higher SI is mainly attributable to its strong antiparasitic efficacy (i.e., low EC_50_ values). A comparative structural analysis reveals that the combination of a nitrofuran moiety with a hydrazone linker is generally favourable (Romero et al., 2017, 2023; Kannigadu et al., 2022), particularly when the hydrazone is attached to a non-aromatic carbonyl moiety, as seen in furazolidone, nitrofurazone and nitrofurantoin. However, nitrofurantoin, which exhibits significantly lower SI, contains a more rigid imidazolidine-2,4-dione ring system featuring two carbonyl groups which may confer lower conformational flexibility that can affect its interaction with biological targets, as well as a more delocalized electronic environment (possible tautomerism). These structural and electronic differences may account for the superior selectivity profile of furazolidone, likely due to its enhanced spatial adaptability and more favourable interaction with biological targets.

The differences found between the antileishmanial effect of nitrofurans and their low-moderate cytotoxic activity should be explained by specific differences in host and pathogen targets. It is well established that nitrofurans are prodrugs that target parasite-specific NTR enzymes to be enzymatically activated (Hall et al., 2011; Wyllie et al., 2012, 2013, 2016; Voak et al., 2013; García-Estrada et al., 2023). Two classes of NTRs have been described: type I NTR (oxygen insensitive) catalyses the reduction of the nitro group in two steps, while type II NTR (oxygen sensitive) catalyses the reduction of the nitro group in a single reduction reaction. The reduction by type I NTR does not generate ROS, as molecular oxygen is not involved as a cosubstrate. In contrast, the reaction carried out by type II NTR generates an unstable nitro radical anion, which is re-oxidized to a nitro group by molecular oxygen that in turn, is converted to a reactive superoxide anion (Wyllie et al., 2013; Voak et al., 2013). In addition to these two types of NTR enzymes, an uncommon NAD(P)H-dependent flavoprotein dubbed NTR2 has been reported to activate the nitroimidazoles delamanid and pretomanid (Patterson et al., 2013; Wyllie et al., 2016). It has been reported that nifurtimox is rapidly converted in trypanosomatids into open chain nitrile metabolite, which is the end product of the multi-step 2-electron reduction by type I NTR and acts as a highly reactive Michael acceptor (Boiani et al., 2010; Vincent et al., 2012; Bot et al., 2013). When tested on intramacrophage amastigotes, it should be considered the possibility that the macrophage could also contribute to any potential metabolization of the nitrofuran drugs through the host's NTR activity. The EC_50_ values provided by Table 3, Table 4 indicate that potency of nitrofurans (except from PYZD-4409 as indicated above) was in the same range for both axenic and intramacrophage amastigotes, thus underscoring the relevance of the parasites' NTR for the selective drug-activation.

It is well documented that, despite their medical properties, nitrofuran derivatives as a class are mutagenic compounds (McCalla, 1983). According to our results in a 384-microwell plate Ames test (Table 5), furazolidone behaved as a more potent mutagen than nitrofurazone in the TA98 and TA100 *S. typhimurium* strains. This effect was previously observed with other *S. typhimurium* strains (Gajewska et al., 1990). These authors suggested that bacterial metabolism may be responsible for the different mutagenic responses produced by nitrofurans in bacteria. In fact, we have shown that the enzymatic activation by mammalian S9 mix prevented the mutagenic effect of furazolidone, thus confirming this hypothesis and previous studies (Ohta et al., 1980). On the contrary, metabolic activation only slightly reduced the mutagenic activity of nitrofurazone, which suggests different mechanisms of mutagenicity for furazolidone and nitrofurazone. Our results indicated that furazolidone induced both frameshift mutations (TA98) and base-pair mutations (TA100) without the addition of S9 mix. On the other hand, nitrofurazone produced base-pair mutations (TA100) regardless of whether it is metabolized or not. For this compound, TA100 was a more sensitive indicator of the mutagenic activity than TA98, as it has been reported for this and other nitrofurans (Goodman et al., 1977).

Some studies have demonstrated that nifurtimox and other nitrofurans do not generate ROS in *Trypanosoma*, but depleted the overall content of intracellular thiols (Boiani et al., 2010; Vincent et al., 2012). In Leishmania parasites, we have observed that nifurtimox and the other nitrofurans under study, were able to induce production of ROS (Fig. 3), thus suggesting additional mechanisms of action. In fact, several molecules of this family of compounds have been reported to behave as inhibitors of TryR (García-Estrada et al., 2023; González-Montero et al., 2024), the key enzyme that maintains the redox balance and manage oxidative stress in trypanosomatids by facilitating the NADPH-dependent reduction of trypanothione, a low-molecular-weight dithiol (N1,N8-bis(glutathionyl)spermidine) unique of trypanosomatids and absent in the mammalian host (Fairlamb et al., 1985; Krauth-Siegel et al., 2003; Manta et al., 2013). Interestingly, the seven nitrofurans tested in this work inhibited TryR (Fig. 4), either in an uncompetitive or competitive manner (Fig. 5).

The inhibitory effect of nitrofurans on TryR is well-known to be dependent on the presence or absence of oxygen. Under low-oxygen conditions, they irreversibly inactivate TryR, while in the presence of oxygen, they act as “subversive” substrates, disrupting the delicate balance of redox reactions, and challenging the parasite's survival. Under aerobic conditions, nitrofurans contribute to the wasteful consumption of NADPH and oxygen, generating free radicals potentially harming the parasite and adding another layer of complexity to their mechanism of action (Henderson et al., 1988). Some nitrofuran molecules previously characterised as “subversive” substrates for trypanosomatids TryR are 2-{5′-nitro (furo-2′-yl)-ethene-1-yl}-4(N,N-diethylamino)-1-methyl-but-1-yl-arninocarbonyl-4-quinoline (chinifur) (Cenas et al., 1994), nifuroxazide, nifuroxime, and nifurprazine (Blumenstiel et al., 1999).

Among the molecules tested in this work, PYZD-4409 exhibited the lowest *K*_i_, suggesting a higher affinity for TryR compared to other nitrofurans, and showed a competitive inhibitory mechanism, like 5-nitro-2-furonitrile and thioridazine (positive control) (Table 6). Competitive inhibitors bind to the active site of the enzyme, preventing the substrate from binding to it. This type of inhibition can be overcome by increasing the substrate concentration, allowing the reaction to proceed. On the contrary, uncompetitive inhibitors bind to the enzyme-substrate complex, reducing the concentration of this complex and thereby, lowering the reaction rate. While competitive inhibition maintains the maximum reaction rate (*V*_max_) and increases the Michaelis constant (*K*_M_), uncompetitive inhibition lowers both *V*_max_ and *K*_M_, potentially enhancing the enzyme's apparent substrate affinity (Strelow et al., 2012). Therefore, whether competitive inhibition is more effective than uncompetitive inhibition can depend on the specific characteristics of the enzyme and the system in which it operates. The fact that furazolidone, nitrofurazone, nitrofurantoin, nifurtimox and 5-nitro-2-furaldehide diacetate behave as uncompetitive inhibitors (Table 6) suggests that these compounds bind to the enzyme-substrate complex of TryR, possibly altering the conformation of the complex in such a way that the catalytic reaction cannot proceed, which could have implications for the redox metabolism of the parasites and, therefore, for their survival and proliferation.

The flexible and site-directed molecular docking study conducted in this work demonstrated that PYZD-4409 exhibits the highest affinity to *L. donovani* TryR – as well as for the three other homologous TryR evaluated – when compared to the other nitrofurans docked (Table 7). This finding aligns well with the lowest *K*_i_ observed for this compound (Table 6). Additionally, the study indicated that PYZD-4409 appears to share – at least partially – the same binding site as the T [S]_2_ substrate, supporting the competitive inhibition mechanism observed for this compound (Table 6). The docking results – including binding modes and energies (Fig. 8 and Table 7) – demonstrated consistent trend across the set of four highly homologous TryRs evaluated in this study. This finding highlights the potential broad-spectrum activity of these nitrofurans as antibiotics and underscore the value of cross-modeling approaches in drug-design targeting trypanosomatids parasites. Moreover, the semi-flexible docking results indicated that furazolidone, nitrofurazone, nitrofurantoin, nifurtimox and 5-nitro-2-furaldehide diacetate preferentially bind within the internal cavity connecting the two T [S]_2_ binding sites rather than occupying the substrate pocket (Fig. 9). Binding to an alternative site distinct from the substrate pocket may, in principle, support the uncompetitive inhibition mechanism observed for these compounds. Notably, furazolidone (Fig. 9, panel c) exhibited the strongest binding affinity among the nitrofurans with uncompetitive inhibition, which is consistent with its best *K*_i_ within this subset of compounds (Table 6) and its potent *in vitro* efficacy reflected by a low EC_50_ (Table 3, Table 4).

In view of these results, where EC_50_ values (either for axenic or intracellular amastigotes) are much lower than *K*_*i*_ values, TryR likely represents a secondary molecular target of the un-metabolized nitrofuran compounds, and it is strongly suggested that the mechanism of action involves other compound species and parasite's (macro)molecules.

## 5 Conclusion

Our findings suggest that furazolidone is the most promising antileishmanial lead candidate among a set of nitrofuran derivatives, based on its combined low *K*_*i*_ (potent inhibitory effect on TryR), low EC_50_ (high *in vitro* cytotoxicity against parasites) and low citotoxity towards mammalian cells, resulting in an excellent SI. These results are consistent with the strongest binding affinity demonstrated by furazolidone in molecular docking, specifically among the nitrofurans exhibiting uncompetitive inhibition. The docking results further support this inhibition mechanism, as furazolidone showed the highest binding affinity when docked at an alternative – but nearby – binding site relative to the substrate pocket. Additionally, the similar binding modes observed for the nitrofurans across a set of four highly homologous TryRs from distinct species of trypanosomatids suggest their potential broad-spectrum antibiotic activity. Further studies are, however, required to confirm these findings and to fully elucidate the mechanism of action and potential of these compounds as therapeutic agents against leishmaniasis.

## CRediT authorship contribution statement

**Julia Andrés-Rodríguez:** Writing – original draft, Visualization, Investigation. **María-Cristina González-Montero:** Investigation. **Nerea García-Fernández:** Investigation. **Juan-José Galano-Frutos:** Writing – review & editing, Writing – original draft, Visualization, Validation, Software, Resources, Methodology, Investigation, Formal analysis, Data curation. **Maria-Cristina de Rosa:** Software, Resources, Methodology, Investigation, Formal analysis, Data curation. **Patricia Ferreira:** Writing – review & editing, Writing – original draft, Visualization, Validation, Software, Resources, Methodology, Investigation, Formal analysis, Data curation. **María-Yolanda Pérez-Pertejo:** Resources, Methodology, Investigation. **Rosa M. Reguera:** Supervision, Resources, Project administration, Methodology, Funding acquisition. **Rafael Balaña-Fouce:** Writing – review & editing, Writing – original draft, Visualization, Validation, Supervision, Resources, Project administration, Methodology, Funding acquisition, Formal analysis, Conceptualization. **Carlos García-Estrada:** Writing – review & editing, Writing – original draft, Visualization, Validation, Supervision, Resources, Project administration, Methodology, Investigation, Funding acquisition, Formal analysis, Conceptualization.

## Institutional review board statement

The animal study protocol was approved by the Ethics Committee of University of León (OEBA 007–2019, approved 25 October 2019).

## Data availability statement

Data supporting the results presented in the article are included in the manuscript. Further inquiries can be directed to the corresponding authors.

## Funding source

This work was supported by Junta de Castilla y León project number LE017G24. María-Cristina González-Montero and Nerea García-Fernández received a fellowship from Universidad de León. Universidad de León has partially funded this research.

## Declaration of competing interest

The authors declare that they have no known competing financial interests or personal relationships that could have appeared to influence the work reported in this paper.
